# Hypersensitivity reactions after femtosecond laser small incision lenticule extraction: a case report of corneal infiltrates

**DOI:** 10.1186/s13223-020-00498-y

**Published:** 2020-11-11

**Authors:** Jiaonan Ma, Lin Zhang, Mengdi Li, Yan Wang

**Affiliations:** 1 Clinical College of Ophthalmology, Tianjin Medical University, No 4. Gansu Road, He-ping District, Tianjin, 300020 China; 2 Tianjin Key Lab of Ophthalmology and Visual Science, Tianjin Eye Hospital, Tianjin Eye Institute, Nankai University Affiliated Eye Hospital, No 4. Gansu Road, He-ping District, Tianjin, 300020 China

**Keywords:** Cornea, Refractive surgery, Contact glass, Hypersensitivity reaction

## Abstract

**Background:**

Femtosecond laser corneal refractive surgery is generally considered safe and effective; however, this procedure is rarely associated with severe allergic reactions. We reported a rare case of hypersensitivity reactions which caused bilateral peripheral corneal infiltrates after femtosecond laser small incision lenticule (SMILE) surgery in a man with a history of fruits allergy.

**Case presentation:**

Here we report the case of a young man who developed white, ring-shaped bilateral peripheral infiltrates that appeared 1 day after an uneventful SMILE surgery. The overlying corneal epithelium was intact; the infiltrate was negative for bacterial culture, but high titers of immunoglobulin E was demonstrated in the blood. Symptomatically, a clinical diagnosis of sterile corneal infiltrates was made, and the patient was treated with topical and systemic steroids. The infiltrates were immunogenic in origin, which may be caused by the contact lenses used for suction duration in surgery. It resolved without corneal scarring in the subsequent months following steroid treatment. The patient’s visual acuity improved.

**Conclusions:**

When patients with a history of allergy who aim to perform corneal refractive surgery, surgeons must consider possible hypersensitivity reactions after treatment. More studies are needed to clarify the relationship between contact glass used in femtosecond laser corneal refractive surgery and IgE mediated hypersensitivity reactions.

## Background

Femtosecond laser small incision lenticule extraction (SMILE), which involves intrastromal lenticule cutting and subsequent lenticule extraction, has emerged as a preferred refractive surgery for myopia correction. As with all ophthalmic procedures, SMILE is associated with certain complications [1, 2]. Infective keratitis is potentially the most sight-threatening complication experienced after SMILE [3]. However, there is no report of peripheral sterile keratitis after SMILE. Incidence of sterile corneal infiltrate has been reported after photorefractive keratectomy (PRK), laser-assisted in situ keratomileusis (LASIK), and corneal crosslinking (CXL) [4–13]. This complication is usually benign, but its diagnosis warrants careful observation. It is easily misdiagnosed as infective keratitis, which is managed differently. Herein, we report a case of bilateral peripheral sterile infiltrate caused by a local immune response that occurred after SMILE, aimed to provide some useful information for the future clinical practice of postoperative complication management of SMILE.

## Case presentation

A 21 year-old man presented to the refractive clinic for surgical evaluation of myopia. His medical history was unremarkable; he had been using glasses for nearsightedness but did not use contact lens. He was highly prone to hypersensitivity and was allergic to various fruits. His uncorrected visual acuity (UCVA) in both eyes was 20/200. Best spectacle-corrected visual acuity (BSCVA) in both eyes was 20/20, with a refraction of − 2.50 − 2.00×5 in the right eye and − 2.25 − 2.00×3 in the left eye. Intraocular pressures (IOPs) of right and left eyes were 16.5 and 16.3 mmHg, respectively. Ocular examination was negative for blepharitis, meibomian gland dysfunction, or other corneal inflammation. The cornea appeared normal, without epithelial defects or infiltrates. Preoperative corneal topography was within the normal limits.

Surgical evaluation indicated bilateral SMILE for myopia correction. Starting 3 days preoperatively, levofloxacin (0.5%; Tarivid, Santen, Inc., Japan) and pranoprofen (5 mL: 5 mg; Senju Pharmaceutical Co. Ltd., Japan) eye drops were instilled 4 times daily. On the day of the surgery, preoperatively, the patient underwent conjunctival sac flushing; his face was prepared and disinfected with compound iodine cotton swab before surgery, and the patient’s face and body was covered with sterile sheets. Oxybuprocaine hydrochloride (0.4%; Benoxil, Santen, Osaka, Japan) eye drops were instilled thrice at 5 min intervals to induce preoperative anesthesia.

SMILE was performed using a 500-kHz VisuMax femtosecond laser system (Carl Zeiss Meditec AG, Jena, Germany) with an S-size contact glass (suction ring) under a negative pressure (30 mmHg). The patient was asked to fixate on a light source before the activation of suction. Laser cutting was performed in the following automated sequence: posterior surface of the lenticule (spiral-in pattern), lenticule side-cut, anterior surface of the lenticule (spiral-out pattern), and finally, and a side-cut on the cap. The laser energy was 140 nJ and intended cap thickness was 120 µm. The diameters of the cap and lenticule were 7.6 mm and 6.6 mm, respectively. A 3.00 mm side-cut was made at the 12-o’clock position for lenticule extraction. After making the lenticule side-cut, a spatula was inserted through the side-cut over the top of the refractive lenticule to separate first, the anterior plane, and subsequently, the posterior plane of the lenticule. Lastly, the lenticule was grasped and extracted through the small incision using micro-forceps. The incision was flushed with balanced salt solution (Alcon Laboratories,Inc., Texas, U.S.A.) after lenticule extraction. Postoperatively, levofloxacin (0.5%) and fluorometholone (0.1%; Flumetholon, Santen, Inc., Japan) were prescribed for instillation, 4 times daily.

**Fig. 1:**
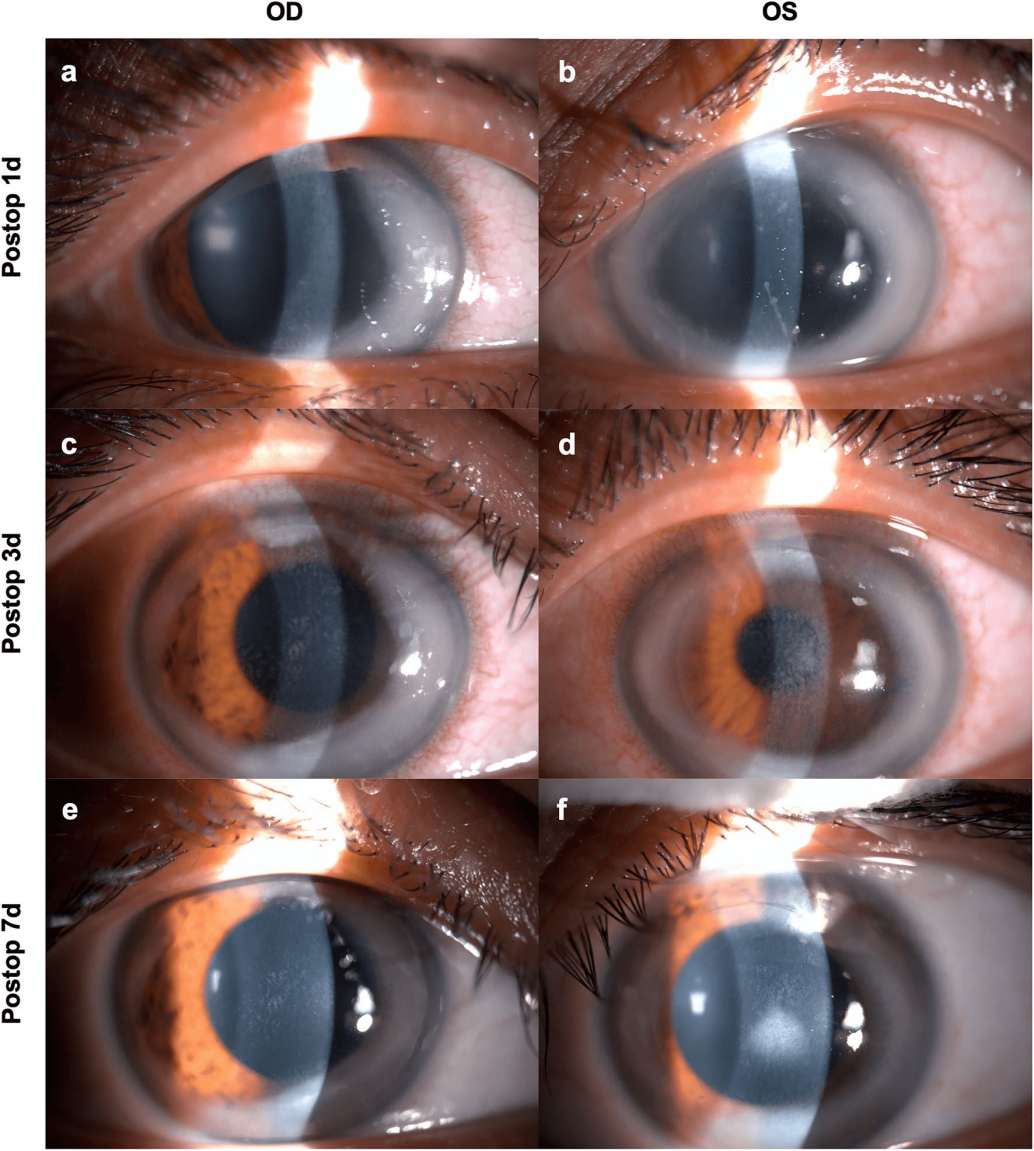
Slit-lamp examination performance at 1, 3 and 7 days after surgery. A complete ring of creamy white infiltrates outside the treatment zone with intact corneal epithelium over the infiltrates and intervening clear zone between the infiltrates and the limbus in both eyes at 1 day after SMILE (**a**, **b**). Creamy white infiltrates began to decrease and stage 1 diffuse lamellar keratitis (DLK) occurred in right eye and stage 2 DLK in left eye at 3 days after SMILE (**c**, **d**). Gray–white peripheral corneal infiltrate from 12 o'clock to 7 o'clock position in both eyes, with decreased DLK in the right eye but increased to stage 4 in the left eye, 7 days after SMILE (**e**, **f**)

One day after SMILE procedure, the patient experienced eye pain, foreign body sensation, and tearing bilaterally. Slit-lamp examination showed a circumferential stromal infiltrate, peripheral to the outside of cap edge, intact corneal epithelium, and an intervening clear zone between the peripheral corneal infiltrate and limbus in both eyes (Fig. 1a, b). There was no anterior chamber reaction. Based on these clinical features, an immune etiology was suspected; however, corneal scraping was not performed. The conjunctival sac secretions were obtained and sent for bacteriological cultures. Blood sample was taken for immunological testing. The patient was initially treated with oral prednisone (60 mg daily), dexamethasone eye drops (once every two hours), and tacrolimus (immunosuppressant; twice daily). Medical examinations were performed every two hours without fail. At 3 o’clock pm on the same day, anterior chamber reaction was noted. Tropicamide phenylephrine eye drops were instilled for pupillary dilation and to ameliorate eye irritation, and sodium hyaluronate eye drops (0.3%; four times daily) was prescribed to relieve dryness and soreness of the eye.

**Fig. 2:**
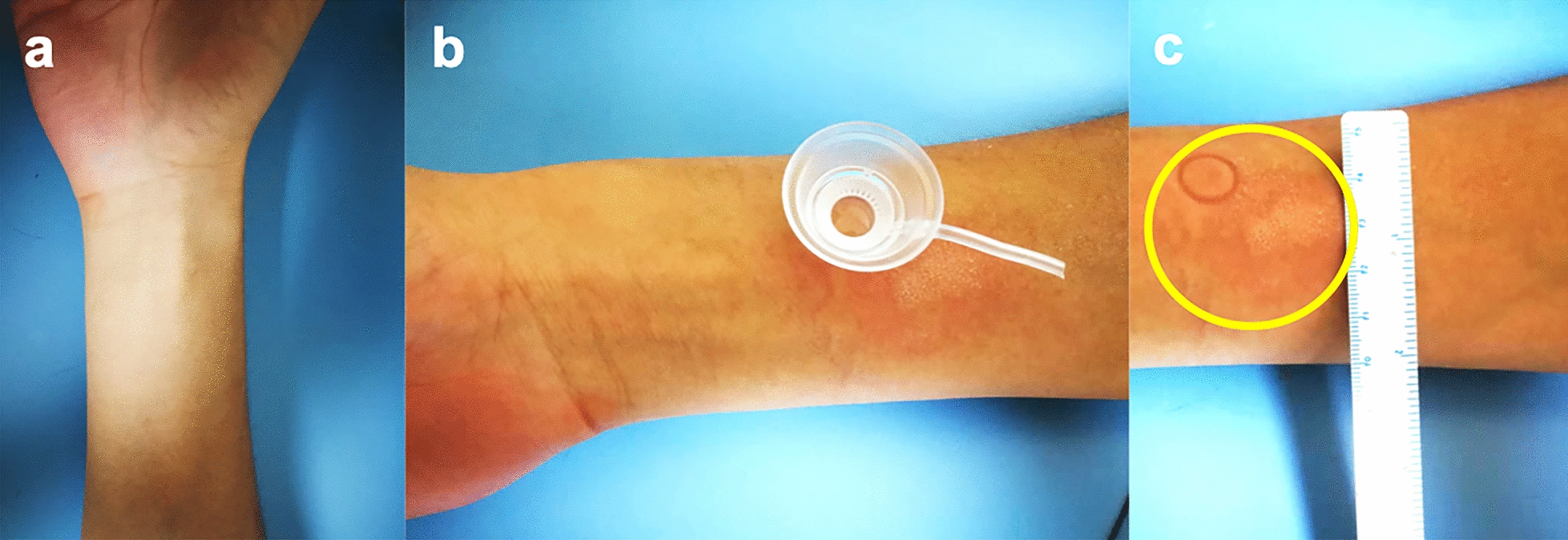
Allergy teat using contact glass. **a** Right forearm of the patient for allergen test. **b** A contact glass was put on the right forearm for allergen test. **c** A positive reaction with redness and swelling

**Fig. 3:**
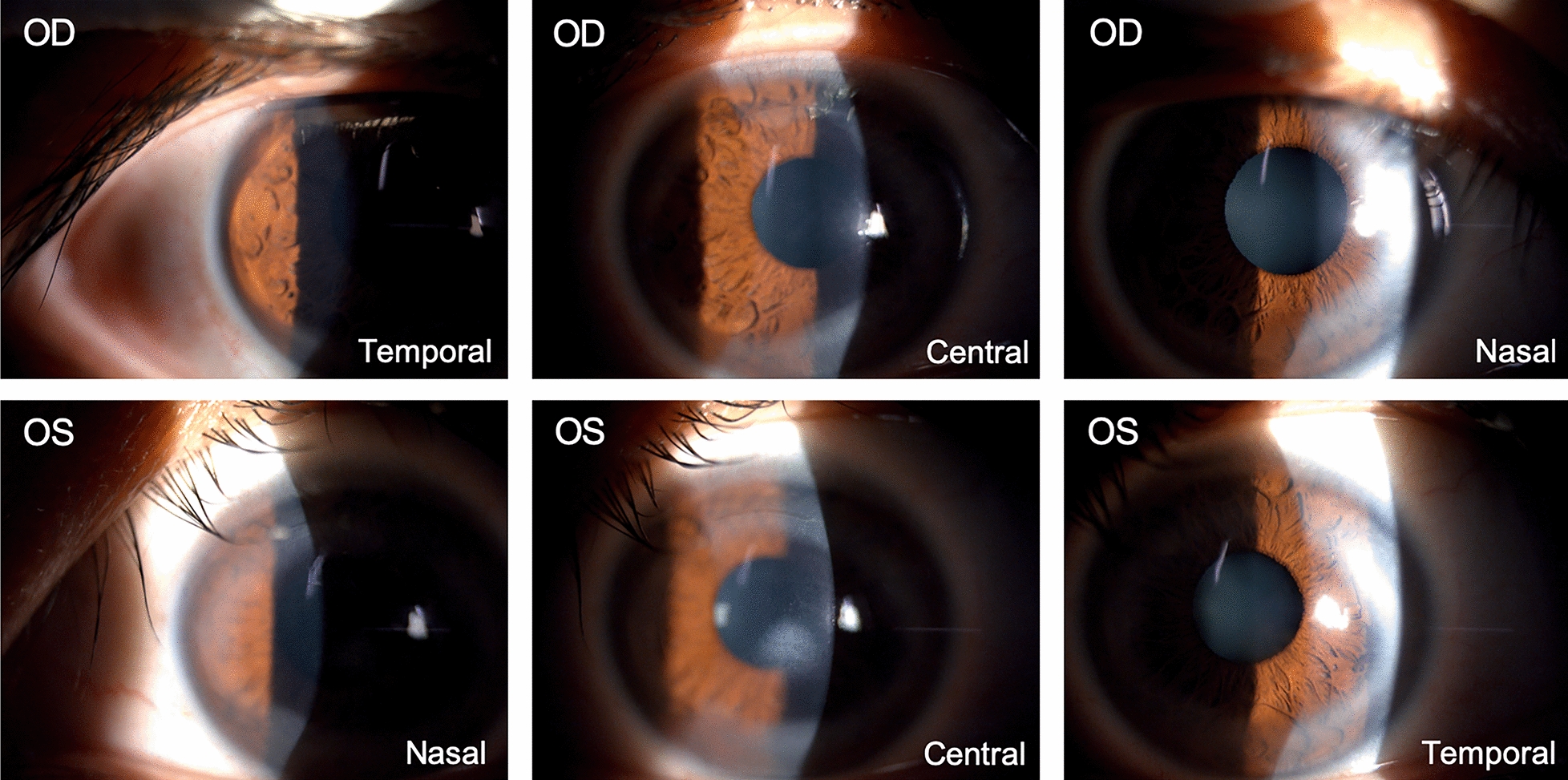
Slit-lamp examination performance at 10 days after surgery. Moderate peripheral corneal infiltrate at temporal, central, and nasal position in both eyes 10 days after SMILE, with healed DLK in the right eye and decreased DLK in the left eye

Two days later, negative results were obtained for bacteriological culture. However, high titers of immunoglobulin E (IgE) with a value of 471 IU/mL was detected in an immunological test of blood. In order to avoid re-invoking the patient's immune response but to further identify the allergen, an allergen test with an applied pressure was conducted after surgery to test for hypersensitivity to the contact glass used during SMILE, the only thing that contacted the corneas. A positive reaction with red and swollen skin was elicited at 10 min (Fig. 2). At 3 days after surgery, the pain persisted and the infiltrates remained intense, with additional presentation of stage 2 diffuse lamellar keratitis (DLK) in the left eye (Fig. 1c, d). However, the density of the infiltrate started to decrease 7 days after treatment initiation despite manifestation of stage 4 DLK in left eye (Fig. 1e, f); Eventually, the drug dosage was tapered for the right eye; the original dosage was retained for use in the left eye. Ten days postoperatively, the density of the infiltrate decreased markedly (Fig. 3). The oral prednisone dosage was tapered gradually and discontinued after 2 weeks; dexamethasone was replaced with prednisolone acetate ophthalmic suspension (1%) and fluorometholone (0.1%) twice daily. Follow-up examination at 1 month showed decreased corneal DLK. However, the IOP readings were high; 18.9 mmHg and 21.6 mmHg in the right and left eyes, respectively. Carteolol (2%) was instilled twice daily to reduce IOP. At 3 months, the DLK disappeared and the IOP readings were 13.4 mmHg and 13.6 mmHg in right and left eyes, respectively.

**Fig. 4:**
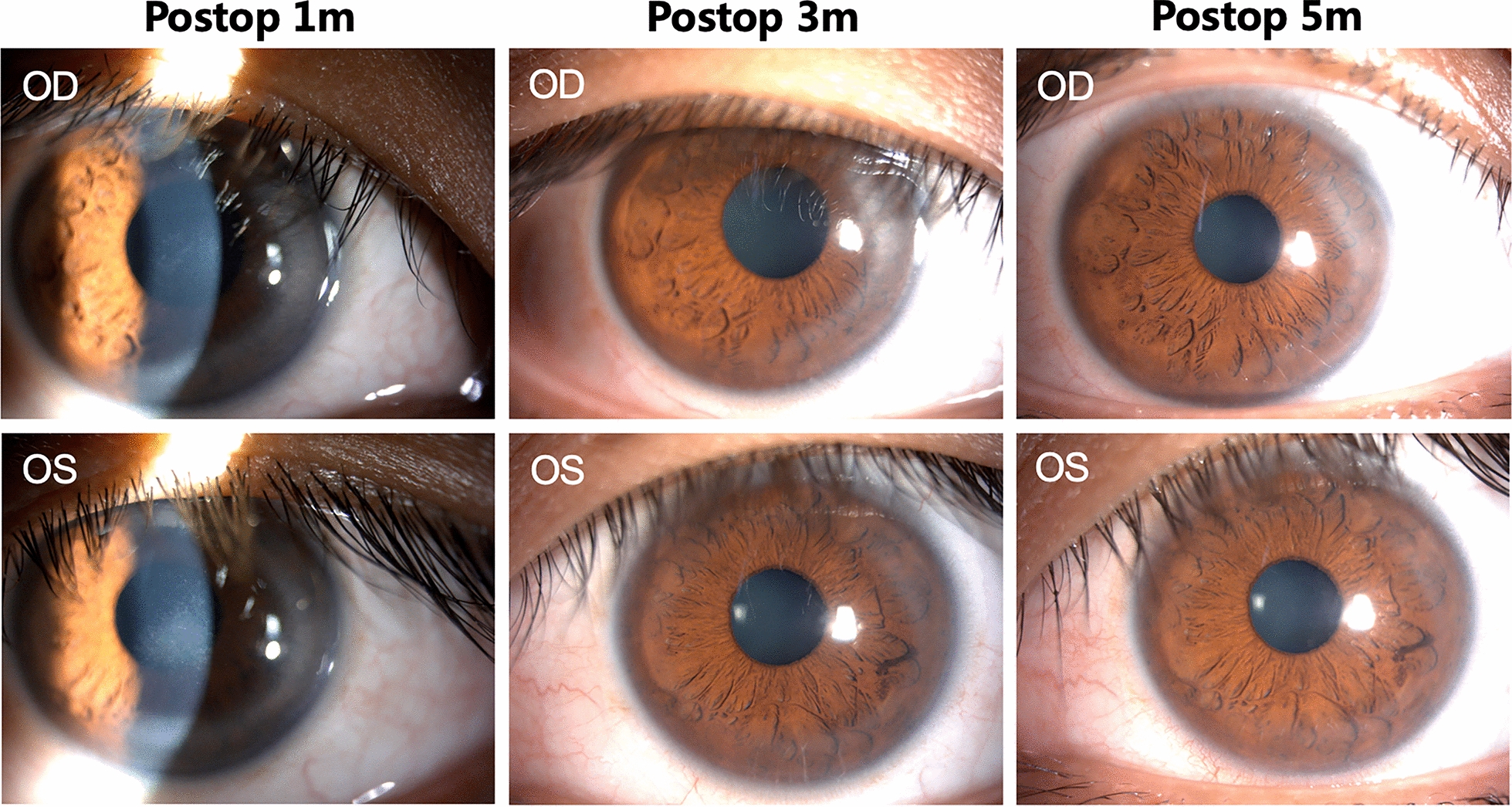
Slit-lamp examination performance at 1, 3 and 5 months after surgery. Moderate peripheral corneal infiltrate with stage 1 DLK in right eye and stage 2 DLK in left eye 1 month after SMILE; Mild peripheral corneal infiltrate at 3 months after SMILE; Healed peripheral corneal infiltrate after 5 months of treatment

By the fifth postoperative month (Fig. 3), the infiltrates resolved completely, and the patient was asymptomatic and maintained a UCVA of 20/25 in both eyes (Fig. 4). The topical treatment was tapered for 1 month. Six months after the procedure, UCVA of 20/20 was maintained in both eyes throughout the postoperative period, and the both corneas were clear, without any scarring. At last follow-up, the patient had no complaint, had normal IOP readings, and no incidence of new pathological sequalae.

## Discussion

To the best of our knowledge, this is the first reported case of sterile corneal infiltrates after SMILE surgery. The patient was diagnosed with bilateral peripheral sterile corneal infiltrates caused by contact glass-induced type I hypersensitivity, based on his proneness to allergies, high level of IgE antibodies in his blood sample, and a positive reaction in the allergen test. However, the etiology of the sterile infiltrates after refractive surgery is still not clear. Use of nonsteroidal anti-inflammatory drugs (NSAIDs) without steroids, usage of bandage contact lenses, immune reaction, topical anesthetic abuse, and laser ablation are reported to be possible causes, while Meibomian gland dysfunction, blepharitis, acne rosacea, psoriasis and hypercholesterolemia are indicated to be important risk factors [4–13].

All culture samples returned without evidence of microorganisms. The condition eventually responded to intensive topical steroid treatment. An allergen test with the contact glass used during SMILE was conducted in this study, showing a positive reaction. The contact glass used in the surgery is composed of a round lens (it is made of glass) and a circular ring (it is made of polyvinyl chloride, PVC). The contact glass is used for suction with a negative pressure of 30 mmHg. While the glass is non immunogenic by its nature, and slit-lamp examination also showed a circumferential stromal infiltrate, peripheral to the outside of cap edge. The morphology of the immune ring in the patient's eye is consistent with the shape of the peripheral ring of contact glass, and the patient had a very strong reaction. Therefore, the peripheral ring was suspected to be the allergen. However, PVC is less likely to be an allergen; we should pay attention to the case with high level of IgE, since negative pressure with this hypersensitive state is likely to trigger an allergic reaction. In addition, the chemical composition of the material used to sterilize the glass /patient interface prepacking, not be excluded completely, should also be paid attention by the manufacturer for safety.

Sterile corneal infiltrates after SMILE present as a localized or circumferential stromal infiltrate peripheral to the cap edge with intact overlying epithelium and an intervening clear zone between the peripheral corneal infiltrate and the limbus, similar to that reported after other refractive surgeries [4–13]. Patients also complain of decreased visual acuity, mild pain, foreign body sensation, and tearing after the first to third postoperative day. However, these symptoms could also be indicative of infectious keratitis, which should be carefully considered in the differential diagnosis because the management is very different, and the prognosis could be disastrous if the infection is not properly treated. Infective keratitis is usually associated with an epithelial defect and an anterior chamber reaction, whereas a sterile infiltrate is associated with a quiet anterior chamber and an intact epithelium. In order to avoid possible complications with invasive investigations such as corneal scraping, the surgeon might decide to just closely monitor the patients’ signs and symptoms. Correct recognition of this benign complication can obviate the need for aggressive interventions. In addition, this surgery is for myopia correction, which is performed on a relatively healthy cornea, and the principle of refractive surgery is to ensure that the patient has a complete and smooth corneal surface for clear vision after surgery. Biopsy is invasive and may cause damage to the patient's cornea, which may affect the recovery after surgery. In future clinical work, corneal biopsy may be taken as appropriate method in the diagnosis and treatment of the disease while ensuring that no further damage is done to the patient.

In addition, sterile related DLK have been described after SMILE surgery [14]. DLK often behaves as a diffuse or peripheral infiltrate that gradually spreads locally to the center of the cornea, but it rarely behaves as reported in this case, which is a peripheral dense annular infiltrate*.* DLK also is characterized by negative culture samples and responding to intensive topical steroid treatment, but its infiltration is limited to the anterior stroma with optical coherence tomography (OCT). In light of the similar presentation with immune response related corneal infiltrates, extreme caution should be taken to include it in the differential diagnosis. In this case, with the clinical presentation and physical signs of the patient, the OCT findings (showing that the depth of infiltration (reached the posterior stroma of the cornea), and the positive immunologic findings, this case is diagnosed as a corneal infiltrate caused by an immune response.

The management of peripheral sterile corneal infiltrates depends on the cause, and if the treatment is appropriate and applied promptly, they usually resolve over time with no longstanding consequences. The most accepted treatment for these infiltrates is the use of topical steroids. Systemic steroids in low doses can also be considered. In this case, the patient had a rapid and severe type I hypersensitivity reaction to the sterile corneal infiltrates, and thus a combination of low dose systemic steroids and topical ocular medication was selected as the initial treatment strategy. When administering high doses of steroids, however, it is necessary to closely monitor the patient's IOP to avoid induction of glucocorticoid-induced glaucoma. At the same time, in the presence of such a strong immune response, the condition of the cornea and the inflammatory reaction in the anterior chamber should be closely monitored, and if necessary, medications to dilate the pupil should be administered.

In the present case, we avoided aggressive corneal scraping and used intensive corticosteroids therapy instead. The corneal transparency recovered completely 5 months after surgery, with an excellent visual outcome. This severe immune response not only adversely affected the patient's life, but also put great psychological pressure on the doctors. Therefore, although it is a very rare postoperative complication, doctors must be aware of the possibility of postoperative occurrence of sterile corneal infiltrates in patients prone to hypersensitivity as well as blepharitis. If such infiltrates do appear, an immunological test should be conducted. It is recommended that doctors pay attention to patients with allergic constitutions. These patients should be advised to conduct an immunological examination before surgery to avoid performing surgery for safety. We also want to draw the attention of immunologists to the cases with immune-related complications following corneal laser surgery.

## Conclusions

The appearance of bilateral peripheral corneal infiltrates after SMILE is an uncommon benign complication, with Type I hypersensitivity being the probable cause. Patients prone to hypersensitivities are at a higher risk of developing these corneal infiltrates. When patients with a history of allergy who aim to perform corneal refractive surgery, surgeons must consider possible hypersensitivity reactions after treatment. More studies are needed to clarify the relationship between contact glass used in femtosecond laser corneal refractive surgery and IgE mediated hypersensitivity reactions.

## Data Availability

The datasets used and analyzed for this case report are available from the corresponding author on reasonable request.

## Abbreviations

SMILE: Small Incision Lenticule Extraction
PRK: Photorefractive Keratectomy
LASIK: Laser-assisted in situ keratomileusis
CXL: Corneal Crosslinking
UCVA: Uncorrected Visual Acuity
BSCVA: Best Spectacle-corrected Visual Acuity
IOP: Intraocular Pressure
DLK: Diffuse Lamellar Keratitis
NSAIDs: Nonsteroidal Anti-inflammatory Drugs
PVC: Polyvinyl Chloride
